# Serum creatinine-to-albumin ratio as a prognostic marker for short- and long-term mortality in critically ill stroke patients: a MIMIC-IV study

**DOI:** 10.3389/fneur.2025.1584368

**Published:** 2025-10-15

**Authors:** Dong Sun, Sichun Chen, Ying Bi, Shuang Wu, Yu Xie, Renwei Zhang, Lei Zhang, Bitang Dan, Huagang Li, Yang Liu, Yumin Liu, Bin Mei, Li Zou

**Affiliations:** ^1^Department of Neurology, Zhongnan Hospital of Wuhan University, Wuhan, Hubei, China; ^2^Economics and Management School of Wuhan University, Wuhan, Hubei, China

**Keywords:** serum creatinine-to-albumin ratio, stroke, critical care, mortality, prognostic biomarker, MIMIC-IV database

## Abstract

**Introduction:**

Stroke remains a leading cause of mortality and disability worldwide, with critically ill patients facing particularly poor outcomes. Existing prognostic markers often fail to capture the full spectrum of metabolic and nutritional disturbances in stroke. The serum creatinine-to-albumin ratio (sCAR), reflecting renal function and nutritional status, may offer improved mortality prediction for the intensive care unit (ICU)-admitted stroke patients.

**Methods:**

This retrospective cohort study used the MIMIC-IV database (v2.2) to analyze 2,819 adult stroke patients admitted to the ICU. Patients were stratified into low- and high-sCAR groups based on an optimal cutoff of 0.419. Predictive performance was assessed using Cox regression, Kaplan–Meier survival analysis, and ROC and RCS curve modeling.

**Results:**

Patients in the high sCAR group (≥0.419) demonstrated significantly higher short- and long-term mortality, including 28-day (31.7% vs. 16.7%, *p* < 0.001) and 1-year mortality (51.0% vs. 27.6%, *p* < 0.001). Multivariate Cox regression confirmed that elevated sCAR was independently associated with increased mortality risk at all endpoints, including 28-day (HR = 2.68, 95% CI: 2.28–3.14, *p* < 0.001) and 1-year (HR = 3.01, 95% CI: 2.61–3.47, *p* < 0.001). ROC analysis showed sCAR outperformed traditional markers, with an AUC of 0.618 for 28-day mortality and 0.639 for 1-year mortality. RCS curves revealed a non-linear association between sCAR and mortality risk, with thresholds indicating elevated risk for both short- and long-term outcomes.

**Conclusion:**

The sCAR is a powerful and clinically relevant biomarker for mortality prediction in critically ill stroke patients. By integrating renal and nutritional assessments, sCAR enhances early risk stratification and supports individualized ICU management.

## 1 Introduction

Stroke is a leading cause of global death and disability, with the Global Burden of Disease Study 2019 identifying it as the second most frequent cause of mortality worldwide (1). Ischemic stroke, constituting approximately 62% of all stroke cases, results from cerebral vessel occlusion, while hemorrhagic stroke arises from vessel rupture and intracerebral hemorrhage (2). Both subtypes can lead to severe complications, including altered consciousness, heightened intracranial pressure, and potentially, multi-organ failure, necessitating intensive monitoring and treatment in the ICU (3).

Reperfusion therapies, such as thrombolysis and thrombectomy, have improved outcomes in acute ischemic stroke (4). However, delayed hospital presentation and limited access to care, especially in low- and middle-income countries (LMICs), hinder timely treatment (5). With the global stroke burden projected to increase disproportionately in LMICs by 2050 (6), simple and reliable risk assessment tools are urgently needed to guide the management of stroke patients without reperfusion therapy.

Serum creatinine (sCr) and albumin (Alb) are staples in the clinical biochemical assessment toolkit. Creatinine levels are a barometer of renal function, while albumin is pivotal in sustaining plasma colloid osmotic pressure and boasts anti-inflammatory, antioxidant, and antithrombotic capabilities (7, 8). Empirical evidence from prior studies has indicated that elevated creatinine or diminished albumin levels are individually linked to an unfavorable prognosis in stroke patients (9, 10). However, the prognostic value of a solitary biomarker is inherently limited; thus, the amalgamation of multiple indicators may bolster prognostic precision.

The serum creatinine-to-albumin ratio (sCAR) has recently emerged as a novel biomarker, reflecting both renal function and nutritional status. Its prognostic value has been demonstrated in various clinical contexts, including acute kidney injury and cardiovascular diseases (11). However, the association between sCAR and short- and long-term all-cause mortality in stroke patients remains unclear. Notably, the majority of prior studies have focused on either ischemic or hemorrhagic stroke, with limited research examining the prognostic utility of sCAR across both subtypes. Given the distinct pathophysiological mechanisms underlying ischemic and hemorrhagic strokes, it is crucial to explore whether sCAR can serve as a unified prognostic indicator for stroke patients admitted to the ICU.

Our study aimed to elucidate the association between sCAR and all-cause mortality at different time points (7-day, 14-day, 21-day, 28-day, 90-day and 1-year) in stroke patients admitted to the ICU. We harness data from the Medical Information Mart for Intensive Care IV (MIMIC-IV, v2.2) database, spanning from 2008 to 2019. By delving into sCAR as a prospective prognostic indicator, our ambition is to shed new light on clinical risk assessment, thereby augmenting the management paradigm for stroke patients.

## 2 Methods

### 2.1 Data source

This study used data from the MIMIC-IV (v2.2) database, a publicly available critical care dataset containing de-identified information from ICU admissions at the Beth Israel Deaconess Medical Center (2008–2019). The database includes demographics, vital signs, laboratory results, treatments, and outcomes. Ethical approval and informed consent were waived as all data were de-identified. Access to the database was authorized after completing the required training on data usage. As described in our previous research (12), the primary investigator, Li Zou (ID: 13349610), authorized to access and extract data from the database was subsequently granted to our research team.

### 2.2 Study population

**Figure 1:**
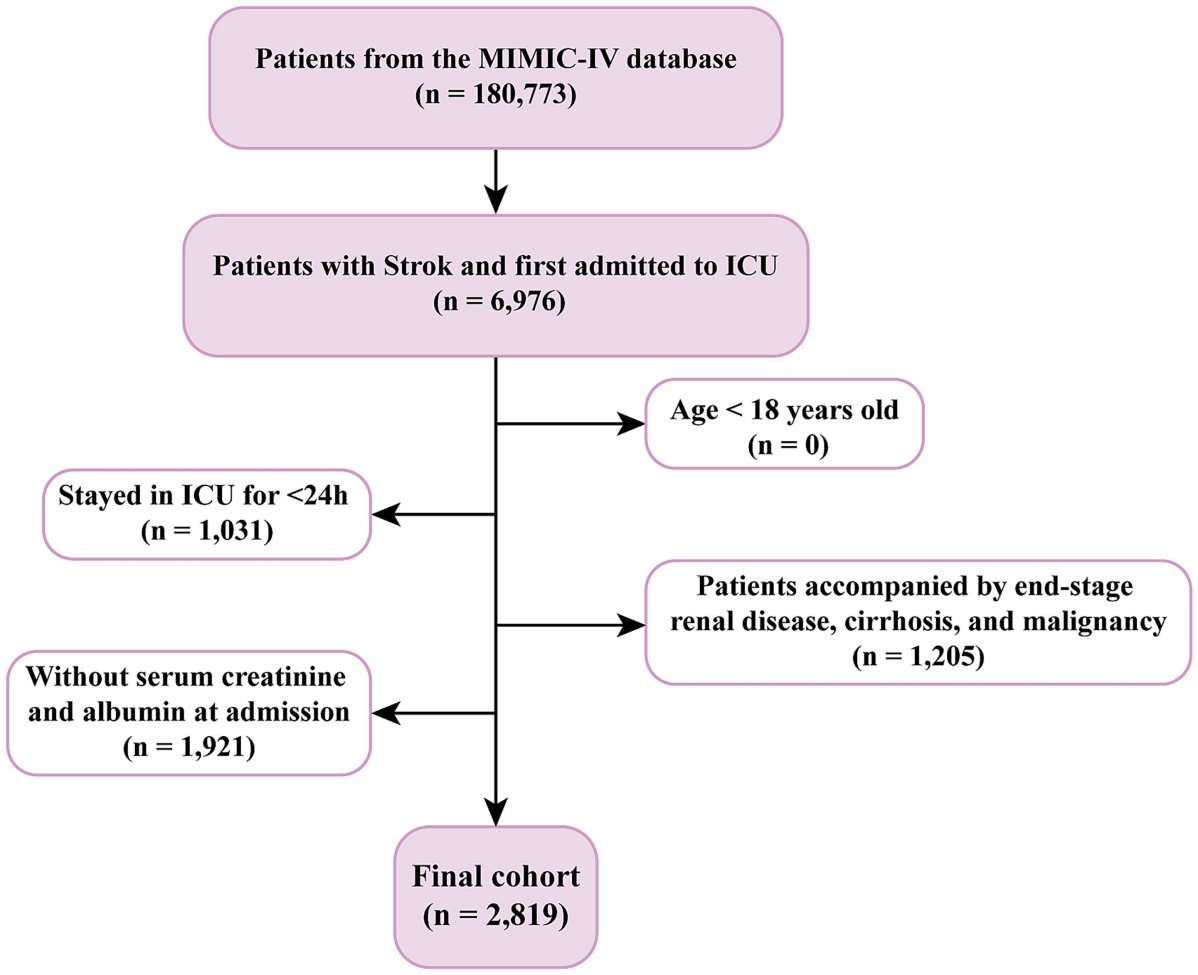
A flowchart for the patient selection process.

For this study, stroke patients were identified using International Classification of Diseases (ICD)-9/10 codes for ischemic (ICD-9: 433–437; ICD-10: I63–I67) and hemorrhagic stroke (ICD-9: 430–432; ICD-10: I60–I62). Inclusion criteria were adult (≥18 years) first-time ICU admissions with sCr and Alb levels measured within 24 h. Exclusion criteria included end-stage renal disease, malignancy, chronic liver disease, missing survival data, or ICU stays <24 h. A total of 2,819 eligible patients were included (Figure 1).

### 2.3 Data extraction

The sCAR was the primary study variable. sCr and Alb levels were measured within 24 h of ICU admission to minimize treatment-related biases. Data were extracted using Structured Query Language (SQL) with PostgreSQL. Extracted variables included demographic information, comorbidities, vital signs, laboratory results, clinical scores [e.g., Sequential Organ Failure Assessment (SOFA)], treatments (e.g., statins), and outcomes. Covariates adjusted for in multivariable Cox regression, including vasopressor drugs and beta blockers, were also extracted and included in the analysis. Supplementary Table 1 summarizes the extracted variables.

### 2.4 Endpoint events

Primary endpoints were all-cause mortality at 7-d, 14-d, 21-d, 28-d, 90-d, and 1-y. Secondary endpoints included ICU length of stay, hospital length of stay, and in-hospital mortality.

### 2.5 Statistical analysis

Continuous variables were summarized as mean ± standard deviation (SD) or median (IQR), depending on their distribution, which was assessed using normality tests (Supplementary Table 2). Categorical variables were summarized as counts and percentages. Group comparisons were conducted using t-tests or ANOVA for normally distributed continuous variables and non-parametric tests for non-normally distributed variables, as well as χ^2^ or Fisher’s exact tests for categorical variables. The treatment of missing data was described as follows: Missing laboratory indicators were imputed using the mean value of the respective variable for the study population. The rate of missing laboratory indicators was less than 5% for all variables included in the analysis. The optimal sCAR cutoff for predicting 28-day mortality was determined using the Youden index. Patients were stratified into low- and high-sCAR groups based on this cutoff. The optimal sCAR cutoff for 28-day mortality was determined using the Youden index. Patients were stratified into low- and high-sCAR groups. Cox proportional hazards models assessed the association between sCAR and mortality, reporting hazard ratios (HRs) and 95% confidence intervals (CIs). Kaplan–Meier curves with log-rank tests compared survival between sCAR groups. ROC analysis evaluated sCAR, sCr, and Alb, reporting sensitivity, specificity, and AUC values. Figures and ROC visualization were added to enhance transparency. Subgroup analyses examined interactions with age, sex, statin use, hypertension, and sepsis. The assumptions for these subgroup analyses were predefined based on clinical relevance and prior evidence suggesting potential interactions. Specifically, we hypothesized that hypertension might modify the predictive ability of sCAR due to its impact on cardiovascular outcomes. Similarly, age, sex, statin use, and sepsis were chosen as they are known to influence stroke prognosis and overall mortality. Figures and ROC visualization were added to enhance transparency. Analyses were conducted using R (v4.2.2).

## 3 Results

### 3.1 Baseline demographic and clinical characteristics

**Figure 2:**
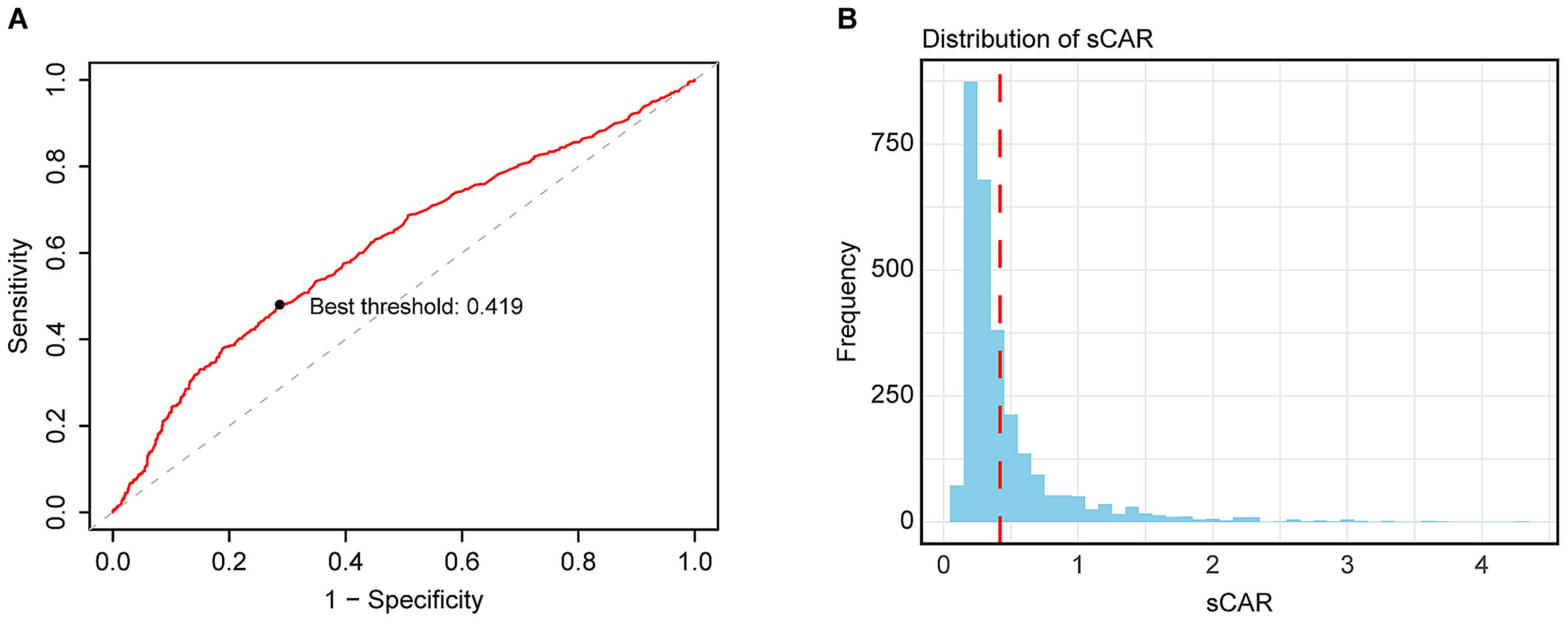
Optimal cutoff point selection: Maximizing risk ratio **(A)** and sCAR ≥ 0.419 distribution **(B)**.

This study included 2,819 ICU-admitted stroke patients (Figure 1), with 51.15% male and 48.85% female. Based on the Youden index, the optimal sCAR cutoff for 28-day mortality was determined to be 0.419 (Figure 2). Patients were stratified into low (<0.419; n = 1,895, 67.19%) and high (≥0.419; *n* = 924, 32.81%) sCAR groups.

Patients in the high sCAR group were older (median age: 72 vs. 70 years, *p* = 0.025), had a higher proportion of males (60.28% vs. 46.70%, *p* < 0.001), and more Black patients (12.12% vs. 8.07%, *p* = 0.003). They also exhibited higher median heart (86 vs. 80 bpm, *p* < 0.001) and respiratory rates (19 vs. 18 breaths/min, *p* < 0.001), but lower systolic (127 vs. 133 mmHg, *p* < 0.001) and diastolic blood pressures (63 vs. 70 mmHg, *p* < 0.001).

The high sCAR group had higher rates of severe comorbidities, including COPD (23.92% vs. 16.78%, *p* < 0.001), sepsis (27.16% vs. 5.86%, *p* < 0.001), and heart failure (42.32% vs. 16.83%, *p* < 0.001). Clinical scores, such as SOFA (5 vs. 3, *p* < 0.001) and the Oxford Acute Severity of Illness Score (OASIS) (35 vs. 31, *p* < 0.001), were significantly worse.

Laboratory markers reflected a more critical state, with higher lactate (1.50 vs. 0.90 mmol/L, *p* < 0.001) and BUN (31 vs. 16 mg/dL, *p* < 0.001), but lower hemoglobin (9.4 vs. 11.4 g/dL, p < 0.001) and albumin (3.2 vs. 3.7 g/dL, *p* < 0.001).

The high sCAR group experienced significantly worse outcomes, including higher ICU (20.02% vs. 8.13%, *p* < 0.001), in-hospital (29.65% vs. 12.40%, *p* < 0.001), 28-day (31.71% vs. 16.73%, *p* < 0.001), and 1-year mortality (50.97% vs. 27.60%, *p* < 0.001). These findings underscore the discriminative power of sCAR stratification for severity and prognosis (Table 1).

**Table 1:** Baseline characteristics of the study population stratified by sCAR groups.

Variables	Total	sCAR		*P*
		< 0.419	≥ 0.419	
	(*n* = 2,891)	(*n* = 1895)	(*n* = 924)	
Demographics				
Age	71 (58–81)	71(57–80)	72 (59–82)	0.025
Gender				<0.001
F	1,377 (48.85)	1,010 (53.3)	367 (39.72)	
M	1,442 (51.15)	885 (46.7)	557 (60.28)	
Race				0.003
Asian	73 (2.59)	55 (2.9)	18 (1.95)	
White	1746 (61.94)	1,180 (62.27)	566 (61.26)	
Black	265 (9.40)	153 (8.07)	112 (12.12)	
Other/unknown	735 (26.07)	507 (26.75)	228 (24.68)	
Vital signs				
Heart rate	82 (71–95)	80.00 (70.00–92.00)	86.00 (74.00–101.00)	<0.001
Respiratory rate	19 (15–22)	18.00 (15.00–22.00)	19.00 (16.00–24.00)	<0.001
SBP	131 (114–149)	133 (117–150)	127 (110–145)	<0.001
DBP	67 (57–80)	70 (58–82)	63.00 (54–76)	<0.001
Temperature	36.83 (36.50–37.22)	36.83 (36.50–37.19)	36.83 (36.44–37.30)	0.468
SpO2	98 (96–100)	98 (96–100)	98 (95–100)	0.008
Comorbidities				
COPD	539 (19.12)	318 (16.78)	221 (23.92)	<0.001
Sepsis	362 (12.84)	111 (5.86)	251 (27.16)	<0.001
HF	710 (25.19)	319 (16.83)	391 (42.32)	<0.001
AF	472 (16.74)	298 (15.73)	174 (18.83)	0.043
Hypertension	1,534 (54.42)	1,202 (63.43)	332 (35.93)	<0.001
Diabetes	853 (30.26)	478 (25.22)	375 (40.58)	<0.001
Clinical treatment				
Vasopressin	217 (7.7)	69 (3.64)	148 (16.02)	<0.001
Statins	1789 (63.46)	1,160 (61.21)	629 (68.07)	<0.001
Beta blockers	2,188 (77.62)	1,419 (74.88)	769 (83.23)	<0.001
MV	1,278 (45.34)	758 (40)	520 (56.28)	<0.001
CRRT	67 (2.38)	1 (0.05)	66 (7.14)	<0.001
ACEI	946 (33.56)	643 (33.93)	303 (32.79)	0.576
Clinical index				
GCS	14 (11–15)	14 (11–15)	14 (11.75–15)	<0.001
SOFA	3 (2–6)	3 (1.5–4)	5 (3–9)	<0.001
SIRS	3 (2–3)	2 (2–3)	3 (2–3)	<0.001
OASIS	33 (27–38)	31 (26–36)	35 (29–41)	<0.001
Laboratory indicators				
WBC	9.90 (7.50–13.10)	9.50 (7.40–12.30)	11.00 (8.00–14.83)	<0.001
RBC	3.58 (3.10–4.16)	3.80 (3.31–4.29)	3.22 (2.82–3.72)	<0.001
Hb	10.70 (9.20–12.30)	11.40 (9.90–12.80)	9.40 (8.30–11.00)	<0.001
PLT	222 (166–302)	226 (176–299)	209 (140.75–308.00)	<0.001
RDW	14.30 (13.30–15.60)	13.90 (13.20–15.10)	15.10 (14.10–16.80)	<0.001
Neutrophil counts	13.79 (7.57–25.87)	12.75 (7.30–25.87)	15.60 (8.74–25.87)	<0.001
Lymphocyte counts	1.96 (0.96–4.45)	2.01 (0.99–4.45)	1.84 (0.92–4.45)	0.152
Eosinophil counts	0.14 (0.03–0.48)	0.14 (0.04–0.48)	0.14 (0.02–0.48)	0.459
BUN	19.00 (13.00–29.00)	16.00 (12.00–21.00)	31.00 (22.00–49.00)	<0.001
eGFR	10.00 (7.00–14.00)	8.00 (7.00–11.00)	15.00 (11.00–19.00)	<0.001
ALT	26.00 (16.00–69.00)	24.00 (15.00–57.00)	33.00 (17.00–76.94)	<0.001
AST	33.00 (21.00–77.00)	29.00 (20.00–60.00)	45.00 (25.00–103.53)	<0.001
TB	0.60 (0.40–0.90)	0.60 (0.40–0.90)	0.65 (0.40–0.90)	0.009
Na	140.00 (137.00–143.00)	140.00 (137.00–143.00)	140.00 (137.00–144.00)	0.013
K	4.00 (3.70–4.30)	3.90 (3.60–4.30)	4.10 (3.70–4.50)	<0.001
Cl	104.00 (100–107)	104.00 (100–107)	105 (100–109)	<0.001
AG	14.00 (12.00–16.00)	14.00 (12.00–16.00)	14.00 (12.00–17.00)	<0.001
TC	163.50 (141.00–180.00)	163.50 (146.00–186.00)	163.50 (131.75–166.00)	<0.001
TG	149.00 (93.05–162.24)	138.00 (88.00–162.24)	162.24 (113.75–162.24)	<0.001
HDL-C	44.57 (39.00–52.00)	44.57 (41.00–55.00)	44.57 (35.00–46.00)	<0.001
LDL-C	90.30 (74.00–103.00)	90.30 (76.00–107.00)	90.30 (67.00–97.00)	<0.001
PT	13.00 (11.90–14.70)	12.80 (11.80–14.00)	13.90 (12.40–16.20)	<0.001
INR	1.20 (1.10–1.30)	1.20 (1.10–1.30)	1.20 (1.10–1.50)	<0.001
APTT	29.60 (26.60–37.10)	29.10 (26.30–34.50)	31.25 (27.00–46.60)	<0.001
Glucose	129 (105.50–165)	126 (103.00–158.00)	136 (110–179)	<0.001
Lactate	1.00 (0.80–1.30)	0.90 (0.70–1.00)	1.50 (1.18–2.10)	<0.001
Cr	1.10 (0.90–1.70)	0.90 (0.80–1.10)	2.10 (1.60–3.20)	<0.001
Alb	3.60 (3.20–4.00)	3.70 (3.40–4.10)	3.20 (2.80–3.60)	<0.001
sCAR	0.31 (0.23–0.50)	0.26 (0.21–0.31)	0.65 (0.51–1.03)	<0.001
Clinical Outcomes				
LOS ICU	3.99 (2.14–8.30)	3.56 (1.97–7.39)	5.20 (2.81–10.89)	<0.001
LOS hospital	11.82 (6.24–21.08)	9.97 (5.56–17.63)	16.80 (8.80–26.85)	<0.001
ICU mortality	339 (12.03)	154 (8.13)	185 (20.02)	<0.001
In-hospital mortality	509 (18.06)	235 (12.4)	274 (29.65)	<0.001
7-day mortality	307 (10.89)	178 (9.39)	129 (13.96)	<0.001
14-day mortality	458 (16.25)	245 (12.93)	213 (23.05)	<0.001
21-day mortality	544 (19.3)	278 (14.67)	266 (28.79)	<0.001
28-day mortality	610 (21.64)	317 (16.73)	293 (31.71)	<0.001
90-day mortality	803 (28.49)	405 (21.37)	398 (43.07)	<0.001
1-year mortality	994 (35.26)	523 (27.6)	471 (50.97)	<0.001

SBP, systolic blood pressure; DBP, diastolic blood pressure; Temp, temperature; SpO2, oxygen saturation; COPD, chronic obstructive pulmonary disease; Sepsis, sepsis; HF, heart failure; AF, atrial fibrillation; MV, mechanical ventilation; CRRT, continuous renal replacement therapy; ACEI, angiotensin-converting enzyme inhibitor; GCS, Glasgow Coma Scale; SOFA, Sequential Organ Failure Assessment; SIRS, systemic inflammatory response syndrome; OASIS, Oxford Acute Severity of Illness Score; WBC, white blood cell; RBC, red blood cell; Hb, hemoglobin; PLT, platelet; RDW, red blood cell distribution width; BUN, blood urea nitrogen; eGFR, estimated glomerular filtration rate; ALT, alanine aminotransferase; AST, aspartate aminotransferase; TB, total bilirubin; Na, serum sodium; K, serum potassium; Cl, serum chloride; AG, anion gap; TC, total cholesterol; TG, triglyceride; HDL-C, high-density lipoprotein cholesterol; LDL-C, low-density lipoprotein cholesterol; PT, prothrombin time; INR, international normalized ratio; APTT, activated partial thromboplastin time; Glucose, glucose; Lactate, lactate; Cr, creatinine; Alb, albumin; sCAR, serum creatinine to albumin ratio; LOS ICU, length of ICU stay; LOS hospital, length of hospital stay.

### 3.2 Univariate and multivariate cox regression models of sCAR and mortality

Cox regression analyses revealed that elevated sCAR (≥0.419) was consistently associated with significantly increased mortality risk across all time points.

In univariate models, high sCAR was linked to higher mortality at 7-day (HR: 2.34, 95% CI: 1.90–2.89), 14-day (HR: 2.52, 95% CI: 2.08–3.06), 21-day (HR: 2.64, 95% CI: 2.22–3.14), 28-day (HR: 2.78, 95% CI: 2.36–3.28), 90-day (HR: 2.95, 95% CI: 2.55–3.42), and 1-year (HR: 3.15, 95% CI: 2.73–3.63; all *p* < 0.001). In multivariate Model 1 (adjusted for age and sex), sCAR remained a robust mortality predictor at all time points (e.g., 28-day HR: 2.68, 95% CI: 2.28–3.14; *p* < 0.001). Model 2, further adjusted for confounders such as vasopressin, beta-blockers, and hematologic parameters, confirmed the independent predictive value of sCAR across all endpoints. Detailed results are presented in Table 2.

**Table 2:** HRs for mortality across different time points by sCAR group in unadjusted and adjusted models.

sCAR	Group	7-d HR (95% CI)	14-d HR (95% CI)	21-d HR (95% CI)	28-d HR (95% CI)	90-d HR (95% CI)	1-y HR (95% CI)
Unadjusted	sCAR < 0.419	1	1	1	1	1	1
	sCAR ≥ 0.419	3.98 (2.14–7.41)	3.78 (2.42–5.91)	3.56 (2.21–5.68)	3.22 (2.01–5.15)	2.84 (1.89–4.26)	2.56 (1.78–3.69)
*P for trend*		0.001	<0.001	<0.001	<0.001	<0.001	<0.001
Model 1	sCAR < 0.419	1	1	1	1	1	1
	sCAR ≥ 0.419	3.45 (1.91–6.58)	3.12 (1.87–5.23)	3.01 (1.68–4.98)	2.89 (1.65–4.54)	2.56 (1.62–4.05)	2.41 (1.62–3.48)
*P for trend*		0.002	<0.001	<0.001	<0.001	<0.001	<0.001
Model 2	sCAR < 0.419	1	1	1	1	1	1
	sCAR ≥ 0.419	3.23 (1.80–6.13)	2.96 (1.77–4.96)	2.89 (1.61–4.89)	2.73 (1.55–4.23)	2.45 (1.51–3.97)	2.29 (1.53–3.32)
*P for trend*		0.004	<0.001	<0.001	<0.001	<0.001	<0.001

HR, Hazard ratios; sCAR, Serum creatinine to albumin.

### 3.3 Kaplan–Meier curve analysis

**Figure 3:**
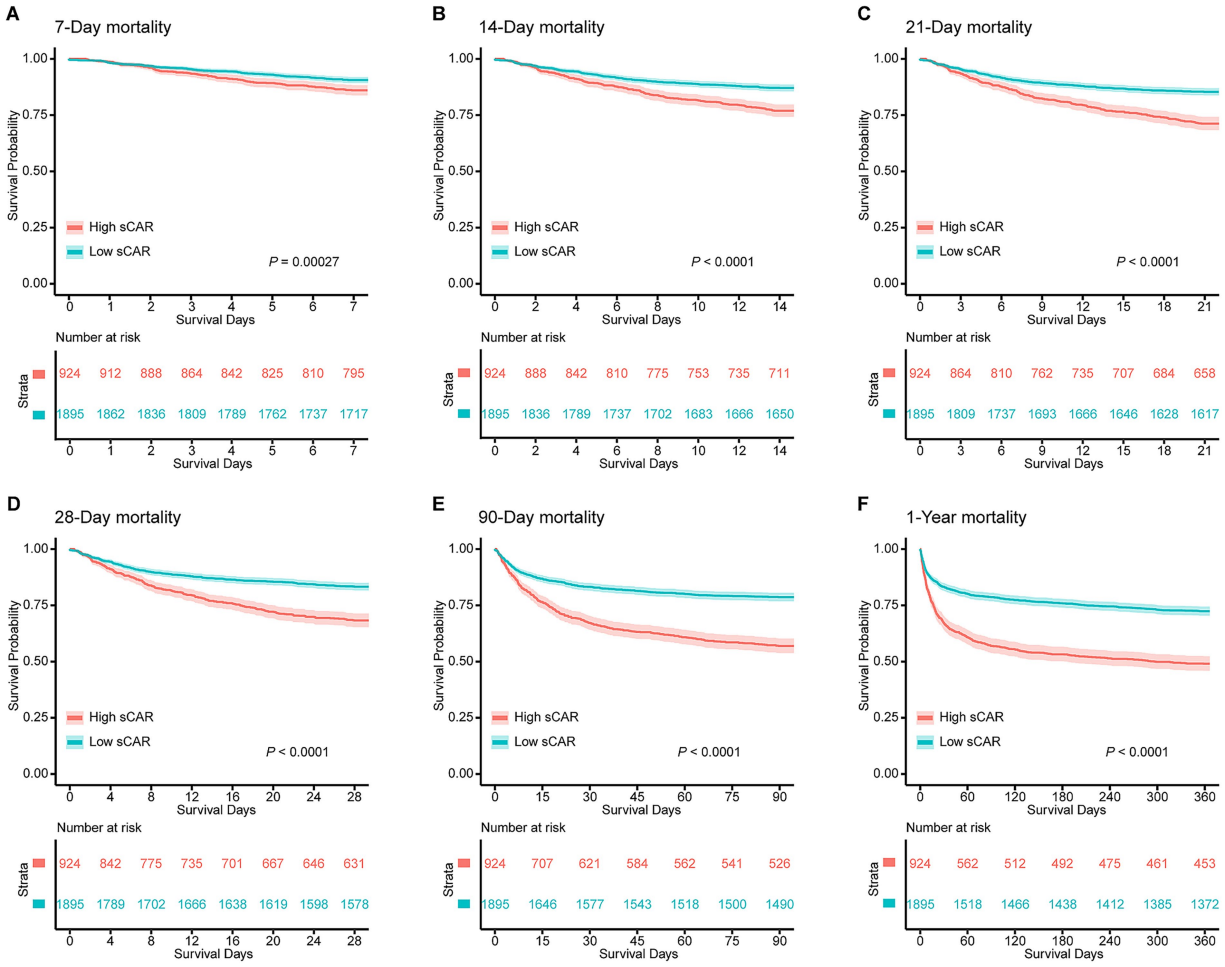
Kaplan–Meier survival analysis curves for all-cause mortality in patients with stroke at 7-d **(A)**, 14-d **(B)**, 21-d **(C)**, 28-d **(D)**, 90-d **(E)**, and 1-y **(F)** of hospital admission.

Kaplan–Meier survival curves demonstrated that patients in the high sCAR group had significantly higher mortality at all assessed time points compared to those in the low sCAR group. Specifically, the cumulative mortality rates in the high sCAR group versus the low sCAR group were 6.1% vs. 1.6% at 7-day (*p* = 0.002), 12.1% vs. 3.7% at 14-day (*p* < 0.001), 15.9% vs. 4.5% at 21-day (*p* < 0.001), 18.2% vs. 5.0% at 28-day (*p* < 0.001), 27.8% vs. 10.2% at 90-day (*p* < 0.001), and 33.3% vs. 14.3% at 1-year (*p* < 0.001). These results highlight the consistent and robust association between elevated sCAR levels and poorer long-term survival outcomes (Figure 3).

### 3.4 ROC and RCS curve analysis

**Figure 4:**
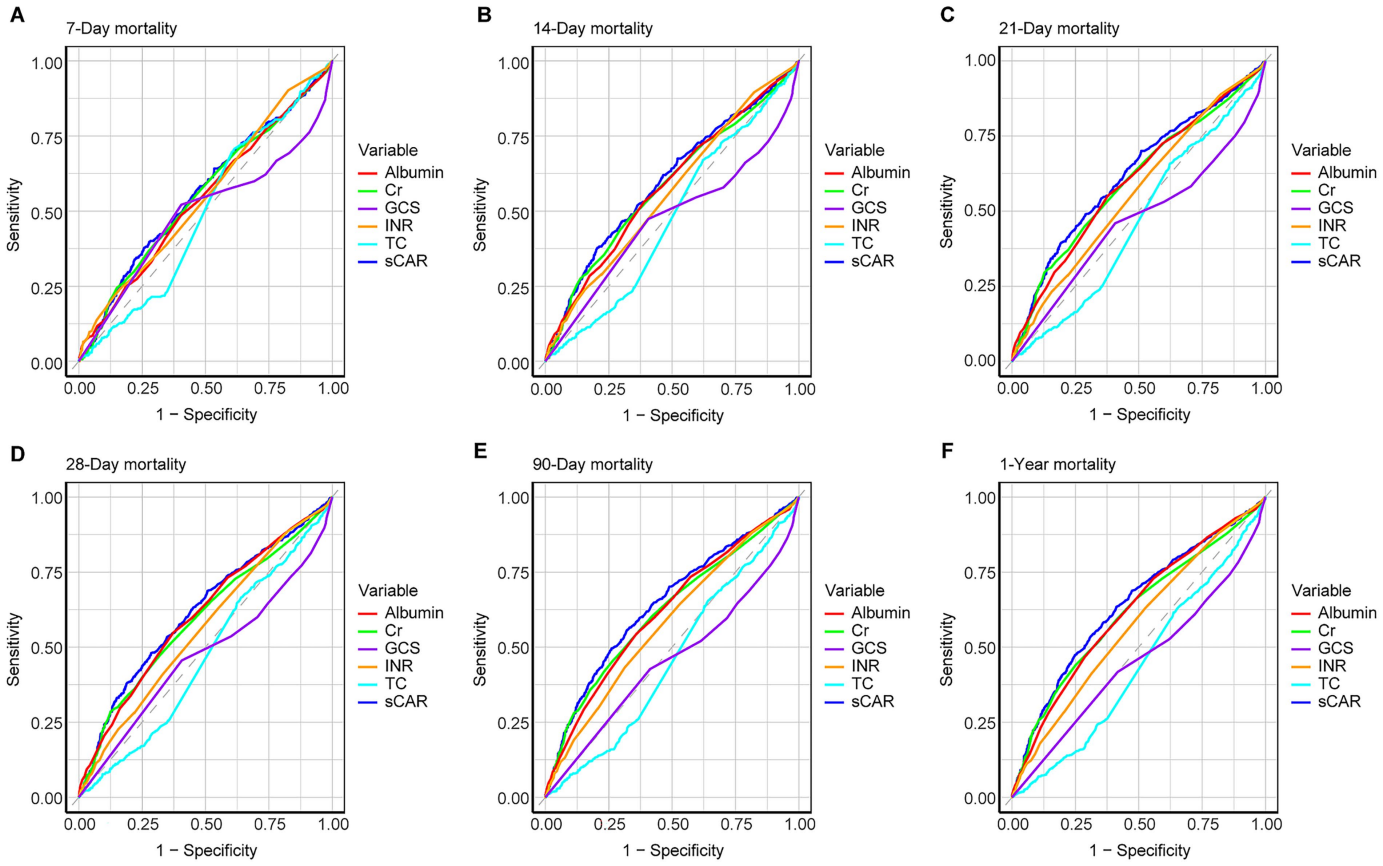
ROC curves for predicting all-cause mortality in patients with stroke at 7-d **(A)**, 14-d **(B)**, 21-d **(C)**, 28-d **(D)**, 90-d **(E)**, and 1-y **(F)** after admission.

ROC analysis assessed the predictive performance of sCAR and other variables (e.g., creatinine, albumin, TC, INR, and GCS) across multiple mortality endpoints (7-day, 14-day, 21-day, 28-day, 90-day, and 1-year). For 28-day mortality, sCAR demonstrated an AUC of 0.618 (95% CI: 0.592–0.644) with a threshold of 0.343 (sensitivity: 57.5%, specificity: 60.3%), while for 1-year mortality, it achieved an AUC of 0.639 (95% CI: 0.617–0.661) with a threshold of 0.315 (sensitivity: 63.4%, specificity: 58.8%). Other variables, such as TC and GCS, had lower AUCs (<0.47 across endpoints). Figure 4 and Table 3 summarize these findings.

**Table 3:** Performance metrics of sCAR and comparative variables for predicting mortality across multiple time points.

Time Point	Variable	AUC	95% CI	Threshold	Sensitivity	Specificity
7-Day mortality	sCAR	0.561	0.526–0.596	0.323	0.570	0.535
	Cr	0.553	0.518–0.588	1.150	0.554	0.537
	Alb	0.540	0.505–0.575	3.450	0.485	0.597
	TC	0.494	0.463–0.525	163.305	0.697	0.396
	INR	0.551	0.517–0.585	1.150	0.603	0.440
	GCS	0.491	0.452–0.530	14.500	0.521	0.596
14-Day mortality	sCAR	0.598	0.569–0.628	0.315	0.620	0.535
	Cr	0.582	0.552–0.612	1.250	0.517	0.617
	Alb	0.582	0.553–0.611	3.450	0.531	0.611
	TC	0.475	0.448–0.502	163.305	0.648	0.392
	INR	0.562	0.533–0.590	1.150	0.627	0.448
	GCS	0.470	0.438–0.501	14.500	0.474	0.594
21-Day Mortality	sCAR	0.621	0.594–0.649	0.378	0.542	0.648
	Cr	0.600	0.572–0.628	1.150	0.596	0.557
	Alb	0.600	0.572–0.627	3.450	0.551	0.622
	TC	0.466	0.441–0.492	160.190	0.658	0.377
	INR	0.562	0.536–0.588	1.150	0.632	0.452
	GCS	0.466	0.437–0.495	14.500	0.460	0.593
28-Day mortality	sCAR	0.618	0.592–0.644	0.343	0.575	0.603
	Cr	0.593	0.566–0.619	1.250	0.521	0.627
	Alb	0.606	0.580–0.632	3.450	0.549	0.626
	TC	0.467	0.443–0.492	160.190	0.648	0.375
	INR	0.559	0.534–0.585	1.150	0.626	0.453
	GCS	0.472	0.445–0.499	14.500	0.456	0.593
90-Day mortality	sCAR	0.643	0.620–0.666	0.371	0.565	0.668
	Cr	0.618	0.594–0.642	1.150	0.603	0.579
	Alb	0.616	0.592–0.639	3.450	0.544	0.641
	TC	0.459	0.436–0.482	160.190	0.636	0.373
	INR	0.580	0.557–0.603	1.150	0.645	0.468
	GCS	0.455	0.431–0.479	14.500	0.427	0.587
1-Year mortality	sCAR	0.639	0.617–0.661	0.315	0.634	0.588
	Cr	0.615	0.592–0.637	1.150	0.589	0.591
	Alb	0.619	0.597–0.641	3.550	0.592	0.583
	TC	0.442	0.420–0.463	160.190	0.616	0.363
	INR	0.573	0.551–0.594	1.150	0.632	0.472
	GCS	0.453	0.431–0.475	14.500	0.418	0.583

AUC, area under the curve; CI, confidence interval; sCAR, serum creatinine to albumin ratio; Cr, creatinine; Alb, albumin; TC, total cholesterol; INR, international normalized ratio; GCS, Glasgow Coma Scale.

**Figure 5:**
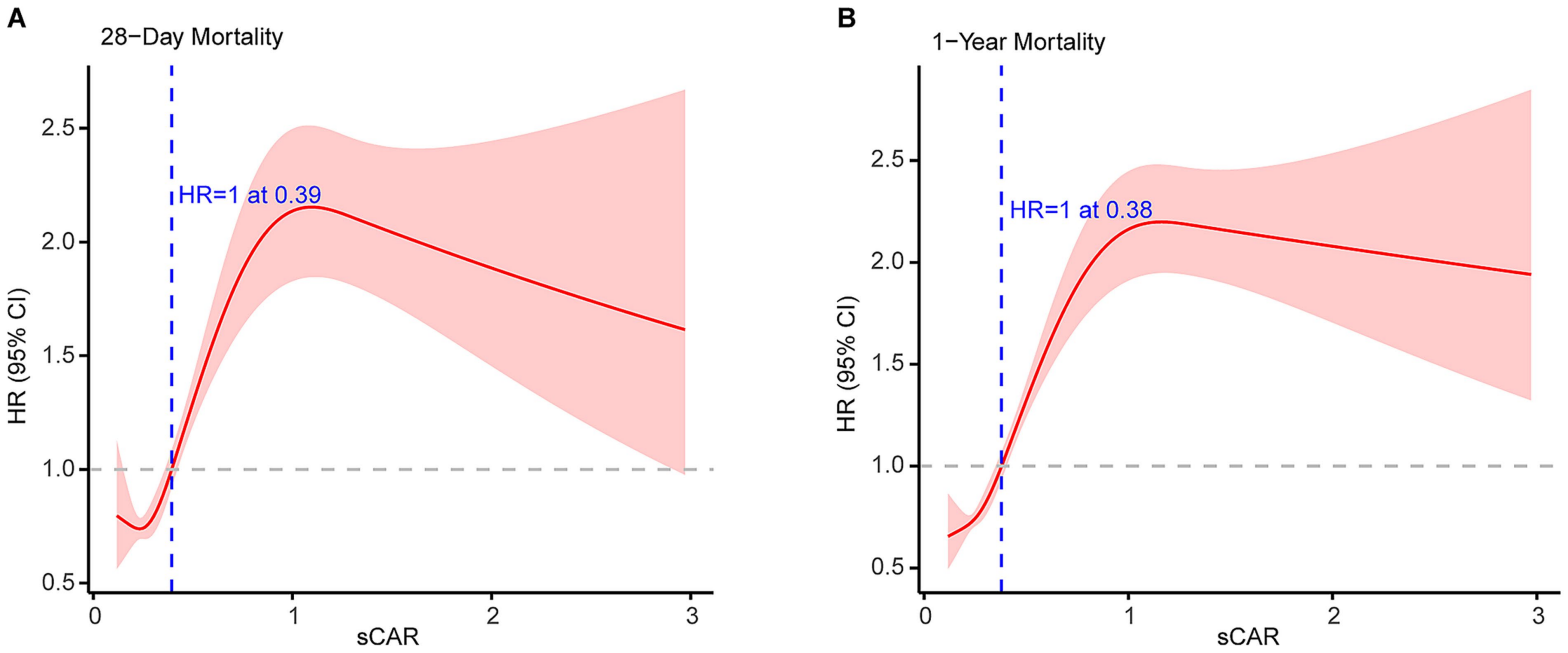
Restricted cubic spline analyses of sCAR for 28-d mortality **(A)** and 1-y mortality **(B)**.

RCS curve analysis further revealed a non-linear association between sCAR and mortality risk (Figure 5). For 28-day mortality, the HR increased sharply beyond a threshold of approximately 0.34, indicating an elevated short-term mortality risk for patients with higher sCAR levels. For 1-year mortality, the HR initially decreased at lower sCAR levels but increased substantially as sCAR continued to rise, with a turning point where HR equals 1 at approximately 0.32. These findings underscore the robust and consistent predictive power of sCAR across both short-term and long-term outcomes.

### 3.5 Subgroup analysis of sCAR and mortality

**Figure 6:**
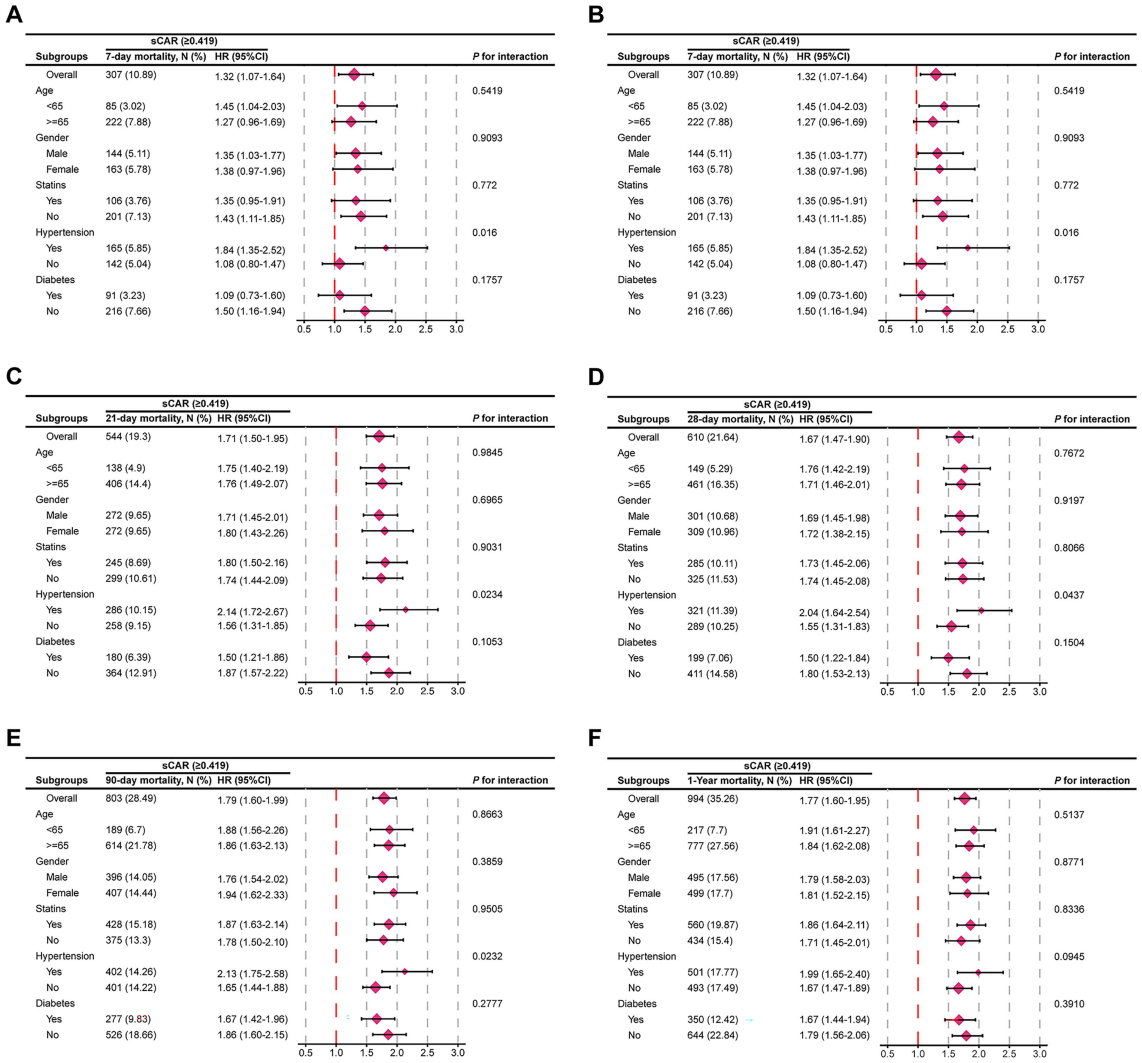
Forest plots of subgroup analysis of the relationship between all-cause mortality and sCAR in patients with stroke admitted 7-d **(A)**, 14-d **(B)**, 21-d**(C)**, 28-d **(D)**, 90-d **(E)**, and 1-y **(F)**.

Figure 6 illustrates the relationship between sCAR and mortality at 7-day, 14-day, 21-day, 28-day, 90-day, and 1-year endpoints across different patient subgroups. Stratified analyses were conducted for age, sex, statin use, hypertension, and diabetes. Most subgroup analyses revealed no significant interaction between sCAR and these factors (*p* > 0.05). However, a significant interaction was observed for hypertension status at the 7-day, 21-day, 28-day, and 90-day endpoints (*P* for interaction = 0.016, 0.023, 0.016, and 0.023, respectively), suggesting that the predictive ability of sCAR may vary based on hypertension status. These findings underscore the potential modifying effect of comorbid hypertension on the prognostic value of sCAR.

## 4 Discussion

In recent years, several studies have explored the role of serum biomarkers, such as the neutrophil-to-lymphocyte ratio (NLR), mean platelets volume (MPV) and the red blood cell distribution width-to-platelet ratio (RPR), in predicting the prognosis of patients with stroke (13–16). However, no study has explored the predictive value of the sCAR in the prognosis of ICU stroke patients. Our study was based on the hypothesis that sCAR, as a composite biomarker reflecting both renal function and nutritional/inflammatory status, would provide a more comprehensive prognostic assessment in critically ill stroke patients compared to individual markers such as creatinine or albumin alone. We provide novel evidence that the sCAR is a robust prognostic marker for predicting both short- and long-term all-cause mortality in critically ill stroke patients admitted to the ICU. Elevated sCAR levels were consistently associated with increased mortality risk at multiple time points, including 7-day, 14-day, 21-day, 28-day, 90-day, and 1-year endpoints. The Kaplan–Meier survival analyses further demonstrated the stark contrast in survival between patients with high versus low sCAR values, underscoring the potential of sCAR as a clinically informative indicator.

Elevated serum creatinine is a well-established indicator of renal dysfunction and is consistently linked to poor outcomes in critically ill stroke patients. Even subtle increases in creatinine within the normal range can elevate stroke risk and overall mortality, independent of traditional vascular risk factors (17, 18). Mechanistically, ischemia–reperfusion injury following a stroke can compromise renal perfusion and precipitate acute kidney injury, contributing to creatinine elevations (19). Systemic inflammation triggered by the stroke may induce endothelial dysfunction and tubular damage, further impairing renal function (20, 21). Oxidative stress, common in ischemic events, can exacerbate renal injury and raise creatinine concentrations (21). In addition, exposure to nephrotoxic agents—such as contrast media and certain antibiotics—can directly harm the kidneys (22, 23). These multifactorial influences, alongside fluid imbalances and metabolic derangements, worsen prognosis by increasing the risk of thrombotic events and functional decline (24–26).

Albumin (Alb), the most abundant plasma protein synthesized by the liver, plays a pivotal role in maintaining plasma oncotic pressure and regulating fluid distribution between compartments (27). As a negative acute-phase reactant, serum Alb levels decrease in response to systemic inflammation, and hypoalbuminemia (commonly defined as <3.5 g/dL) has emerged as a significant prognostic marker in a variety of conditions, including stroke (8, 28, 29). Beyond its structural and transport functions, Alb displays antioxidant, anti-inflammatory, and antithrombotic properties, contributes to endothelial integrity, and modulates platelet activation and aggregation (30–32). In ischemic stroke, low Alb levels have been linked to increased short-term and long-term mortality, as well as poor functional recovery (29). This relationship may be explained by albumin’s role in countering oxidative stress, mitigating endothelial dysfunction, and reducing vascular permeability—key mechanisms that, when compromised, can exacerbate edema, neuronal injury, and overall disease severity (33, 34). Alb serves as an important prognostic indicator, however, it is influenced by factors such as nutritional status, hepatic function, and systemic disease burden, limiting its specificity in stroke prediction (33).

Despite their individual prognostic relevance, relying on creatinine or Alb alone may be insufficient due to the multifactorial nature of critical illnesses. The sCAR metric combines creatinine and Alb into a single measure, potentially offering a more comprehensive reflection of renal, nutritional, and systemic health. By integrating two complementary biomarkers, sCAR demonstrates greater specificity and predictive power for risk stratification compared to either variable alone. This study supports sCAR as a valuable tool for assessing mortality risk in critically ill stroke patients.

Our findings underscore the clinical utility of sCAR as a robust biomarker for predicting short- and long-term mortality in ICU-admitted ischemic stroke patients. Unlike individual markers like creatinine or Alb, sCAR integrates renal function and nutritional/inflammatory status, providing a more comprehensive physiological assessment. Its superior prognostic accuracy, compared to traditional scoring systems like SOFA or GCS, reflects its ability to capture the complex interplay of neurovascular, metabolic, and inflammatory factors in critical illness (35, 36). However, the reported AUC values for sCAR (0.618–0.639) hold practical significance in clinical practice, as they outperform traditional markers and provide a more nuanced understanding of patient risk. RCS analyses further highlight sCAR’s predictive power, identifying thresholds beyond which mortality risk escalates. Such insights enable precise risk stratification and early interventions, including optimized fluid management, nutritional support, and vigilant monitoring for renal or inflammatory complications, aligning with evidence that early metabolic optimization improves outcomes (37–39).

Additionally, Subgroup analyses confirmed sCAR’s prognostic value across various subgroups, including those stratified by age, sex, statin use, hypertension, and diabetes. Hypertension significantly modified sCAR’s predictive capacity at several time points, with heightened risk observed in hypertensive patients. These findings highlight the potential for sCAR to guide personalized therapeutic strategies in neurocritical care (40, 41). Interestingly, our findings indicated a higher HR for sCAR in non-diabetic patients compared to diabetic patients, despite diabetic patients having a higher overall mortality rate (41.03% vs. 32.76%). This paradoxical observation likely reflects differences in baseline hazard between the two groups: diabetic patients inherently exhibit a higher baseline mortality risk, potentially attenuating the relative impact of sCAR. In contrast, non-diabetic patients, with lower baseline risk, experienced a more pronounced effect of sCAR on mortality. Importantly, interaction analysis revealed no significant modifying effect of diabetes on the relationship between sCAR and mortality (*P* for interaction = 0.3898), supporting the consistency of sCAR’s prognostic value across diabetes subgroups. These results underscore the importance of considering baseline risk in interpreting HRs and provide further insight into sCAR’s nuanced role in stroke prognosis.

Fluid management is crucial in stroke patients, especially when addressing dehydration and its associated risks. While dehydration therapies, such as mannitol or diuretics, are commonly employed to reduce cerebral edema and intracranial pressure, they may exacerbate renal dysfunction, particularly in patients with elevated sCAR, indicating compromised renal function and nutritional status (42, 43). Proper fluid management strategies, including close monitoring of renal function and individualized hydration plans, are essential to mitigate these risks and optimize outcomes.

Alb plays a vital role in fluid management for stroke patients, especially those with hypoalbuminemia. Its colloid properties enhance oncotic pressure, restore circulating volume, and provide antioxidant and anti-inflammatory benefits, potentially reducing tissue injury, cerebral edema, and intracranial pressure (27, 44). While albumin administration may improve neurological outcomes in some cases, overuse risks fluid overload, necessitating individualized treatment plans (45, 46). In patients with elevated sCAR, optimizing fluid management to maintain renal perfusion and address hypoalbuminemia is critical. Future studies should explore the relationship between sCAR and fluid management strategies, as well as their effects on neurological and systemic outcomes.

This study’s strength lies in its use of the large and well-characterized MIMIC-IV database, which enhances the generalizability of our findings (47). Comprehensive analytical methods, including Cox regression, Kaplan–Meier analyses, and restricted cubic spline modeling, provide robust validation of sCAR’s prognostic value (48, 49). However, several limitations should be acknowledged. First, the retrospective, single-center design limits external validity, and evolving clinical practices over the study period may introduce heterogeneity (40).

The most significant limitation is the lack of stroke-specific data, an inherent constraint of the MIMIC-IV database. We were unable to account for crucial prognostic factors such as stroke severity (e.g., NIHSS scores), stroke subtype (ischemic vs. hemorrhagic), or detailed neuroimaging findings (50). This absence undoubtedly limits the internal validity of our results and their generalizability to all stroke populations. Consequently, our use of all-cause mortality, while an objective endpoint, precludes analysis of neurologic-specific outcomes such as functional recovery. Thus, the prognostic value of sCAR identified here should be interpreted as its association with overall physiological reserve and mortality risk in a general cohort of ICU-admitted stroke patients, rather than as a specific biomarker of neurologic injury severity.

Future studies are warranted to incorporate stroke severity scores and imaging data to provide a more nuanced understanding of sCAR’s predictive capacity and to determine if its utility varies across stroke subtypes. Prospective, multicenter cohorts are needed to validate sCAR and explore its correlation with functional outcomes. Furthermore, a detailed mechanistic explanation of how renal dysfunction and hypoalbuminemia synergistically influence stroke prognosis is crucial. Integrating sCAR with existing risk stratification tools may enhance its predictive capacity, ultimately guiding individualized management strategies for critically ill stroke patients.

## 5 Conclusion

The sCAR is a strong independent predictor of short- and long-term all-cause mortality in critically ill stroke patients. By integrating renal function and nutritional/inflammatory status, sCAR offers superior prognostic value compared to creatinine or albumin alone. These results highlight its potential to improve risk stratification and guide clinical decisions in the ICU. Prospective multicenter studies are warranted to confirm these findings and explore sCAR’s broader role in neurocritical care.

## Data Availability

Publicly available datasets were analyzed in this study. This data can be found here: direct Link to the data: https://physionet.org/content/mimiciv/2.2/, Repository Name: PhysioNet, Accession Number: MIMIC-IV v2.2.
