# Supplementary material for: Serum creatinine-to-albumin ratio as a prognostic marker for short- and long-term mortality in critically ill stroke patients: a MIMIC-IV study
Source: Front Neurol. 2025 Oct 15;16:1584368. doi: 10.3389/fneur.2025.1584368 (PMC12568402; doi:10.3389/fneur.2025.1584368)
Supplement: Supplementary file 1 [file Table_1.docx]

| **Supplementary Table 1. Covariates extracted in detail from the MIMIC-IV database** | |
| --- | --- |
| **Items** | **Composition** |
| **Demographics** | Age, Gender (F, M), Race (Asian, White, Black, Other/Unknown) |
| **Vital Signs** | Heart rate, Respiratory rate, Systolic blood pressure (SBP), Diastolic blood pressure (DBP), Oxygen saturation (SpO2) |
| **Comorbidities** | Chronic obstructive pulmonary disease (COPD), Sepsis, Heart failure (HF), Atrial fibrillation (AF), Hypertension, Diabetes |
| **Clinical Treatment** | Vasopressin, Statins, Beta blockers, Mechanical ventilation (MV), Continuous renal replacement therapy (CRRT), Angiotensin-converting enzyme inhibitor (ACEI) |
| **Clinical Index** | Glasgow Coma Scale (GCS), Sequential Organ Failure Assessment (SOFA), Systemic inflammatory response syndrome (SIRS), Oxford Acute Severity of Illness Score (OASIS) |
| **Laboratory Indicators** | White blood cell (WBC), Red blood cell (RBC), Hemoglobin (Hb), Platelet (PLT), Red blood cell distribution width (RDW), Neutrophil counts, Lymphocyte counts, Eosinophil counts, Blood urea nitrogen (BUN), Estimated glomerular filtration rate (eGFR), Alanine aminotransferase (ALT), Aspartate aminotransferase (AST), Total bilirubin (TB), Serum sodium (Na), Serum potassium (K), Serum chloride (Cl), Anion gap (AG), Total cholesterol (TC), Triglyceride (TG), High-density lipoprotein cholesterol (HDL-C), Low-density lipoprotein cholesterol (LDL-C), Prothrombin time (PT), International normalized ratio (INR), Activated partial thromboplastin time (APTT), Glucose, Lactate, Creatinine (Cr), Albumin (Alb), Serum creatinine to albumin ratio (sCAR) |
| **Clinical Outcomes** | Length of ICU stay (LOS ICU), Length of hospital stay (LOS hospital), ICU mortality, In-hospital mortality, 7-day mortality, 14-day mortality, 21-day mortality, 28-day mortality, 90-day mortality, 1-year mortality |
