# Supplementary material for: Serum creatinine-to-albumin ratio as a prognostic marker for short- and long-term mortality in critically ill stroke patients: a MIMIC-IV study
Source: Front Neurol. 2025 Oct 15;16:1584368. doi: 10.3389/fneur.2025.1584368 (PMC12568402; doi:10.3389/fneur.2025.1584368)
Supplement: Supplementary file 2 [file Table_2.docx]

**Supplementary Table 1. Test of normality results of continuous variables**

| **Variable** | **Statistic*** | ***P* value*** | **Normality** |
| --- | --- | --- | --- |
| Age | 0.9569735 | 4.24E-28 | Not Normal |
| Heart rate | 0.9789623 | 5.16E-20 | Not Normal |
| Respiratory rate | 0.3277985 | 8.74E-73 | Not Normal |
| SBP | 0.9800738 | 1.85E-19 | Not Normal |
| DBP | 0.9514401 | 1.31E-29 | Not Normal |
| Temperature | 0.1857034 | 1.04E-76 | Not Normal |
| SpO2 | 0.73821 | 5.16E-55 | Not Normal |
| GCS | 0.761404 | 2.10E-53 | Not Normal |
| SOFA | 0.8970812 | 6.22E-40 | Not Normal |
| SIRS | 0.8989886 | 1.18E-39 | Not Normal |
| OASIS | 0.9927109 | 1.03E-10 | Not Normal |
| WBC | 0.7369468 | 4.25E-55 | Not Normal |
| RBC | 0.9890855 | 7.03E-14 | Not Normal |
| Hb | 0.9887844 | 4.14E-14 | Not Normal |
| PLT | 0.9019304 | 3.20E-39 | Not Normal |
| RDW | 0.8590668 | 9.88E-45 | Not Normal |
| Neutrophil counts | 0.7448342 | 1.45E-54 | Not Normal |
| Lymphocyte counts | 0.6183964 | 8.27E-62 | Not Normal |
| Eosinophil counts | 0.3377075 | 1.74E-72 | Not Normal |
| BUN | 0.7589721 | 1.41E-53 | Not Normal |
| eGFR | 0.8122536 | 2.34E-49 | Not Normal |
| ALT | 0.1436437 | 9.27E-78 | Not Normal |
| AST | 0.1114584 | 1.56E-78 | Not Normal |
| TB | 0.2713696 | 2.02E-74 | Not Normal |
| Na | 0.96136 | 8.62E-27 | Not Normal |
| K | 0.961071 | 7.01E-27 | Not Normal |
| Cl | 0.9760043 | 2.17E-21 | Not Normal |
| AG | 0.4161341 | 5.74E-70 | Not Normal |
| TC | 0.7899825 | 3.14E-51 | Not Normal |
| TG | 0.522533 | 4.82E-66 | Not Normal |
| HDL-C | 0.9168315 | 7.69E-37 | Not Normal |
| LDL-C | 0.8943358 | 2.53E-40 | Not Normal |
| PT | 0.3465822 | 3.26E-72 | Not Normal |
| INR | 0.3400815 | 2.06E-72 | Not Normal |
| APTT | 0.6216957 | 1.20E-61 | Not Normal |
| Glucose | 0.7478163 | 2.32E-54 | Not Normal |
| Lactate | 0.6600656 | 1.11E-59 | Not Normal |
| Cr | 0.6565197 | 7.18E-60 | Not Normal |
| Alb | 0.9891358 | 7.69E-14 | Not Normal |
| sCAR | 0.6357986 | 6.04E-61 | Not Normal |
| LOS ICU | 0.7089923 | 7.01E-57 | Not Normal |
| LOS hospital | 0.7510899 | 3.90E-54 | Not Normal |

*: Kolmogorov-Smirnov Test

SBP, systolic blood pressure; DBP, diastolic blood pressure; SpO2, oxygen saturation; COPD, chronic obstructive pulmonary disease; HF, heart failure; AF, atrial fibrillation; MV, mechanical ventilation; CRRT, continuous renal replacement therapy; ACEI, angiotensin-converting enzyme inhibitor; GCS, Glasgow Coma Scale; SOFA, Sequential Organ Failure Assessment; SIRS, systemic inflammatory response syndrome; OASIS, Oxford Acute Severity of Illness Score; WBC, white blood cell; RBC, red blood cell; Hb, hemoglobin; PLT, platelet; RDW, red blood cell distribution width; BUN, blood urea nitrogen; eGFR, estimated glomerular filtration rate; ALT, alanine aminotransferase; AST, aspartate aminotransferase; TB, total bilirubin; Na, serum sodium; K, serum potassium; Cl, serum chloride; AG, anion gap; TC, total cholesterol; TG, triglyceride; HDL-C, high-density lipoprotein cholesterol; LDL-C, low-density lipoprotein cholesterol; PT, prothrombin time; INR, international normalized ratio; APTT, activated partial thromboplastin time; Cr, creatinine; Alb, albumin; sCAR, serum creatinine to albumin ratio; LOS ICU, length of ICU stay; LOS hospital, length of hospital stay
